# Functional Characterization of Arabidopsis PHL4 in Plant Response to Phosphate Starvation

**DOI:** 10.3389/fpls.2018.01432

**Published:** 2018-10-01

**Authors:** Zhen Wang, Zai Zheng, Li Song, Dong Liu

**Affiliations:** MOE Key Laboratory of Bioinformatics, Center for Plant Biology, School of Life Sciences, Tsinghua University, Beijing, China

**Keywords:** PHL4, PHR1, Pi starvation responses, functional redundancy, plant development

## Abstract

Plants have evolved an array of adaptive responses to cope with phosphate (Pi) starvation. These responses are mainly controlled at the transcriptional level. In Arabidopsis, PHR1, a member of the MYB-CC transcription factor family, is a key component of the central regulatory system controlling plant transcriptional responses to Pi starvation. Its homologs in the MYB-CC family, PHL1 (PHR1-LIKE 1), PHL2, and perhaps also PHL3, act redundantly with PHR1 to regulate plant Pi starvation responses. The functions of PHR1’s closest homolog in this family, PHL4, however, have not been characterized due to the lack of its null mutant. In this work, we generated two *phl4* null mutants using the CRISPR/Cas9 technique and investigated the functions of PHL4 in plant responses to Pi starvation. The results indicated that the major developmental, physiological, and molecular responses of the *phl4* mutants to Pi starvation did not significantly differ from those of the wild type. By comparing the phenotypes of the *phr1* single mutant and *phr1phl1* and *phr1phl4* double mutants, we found that PHL4 also acts redundantly with PHR1 to regulate plant Pi responses, but that its effects are weaker than those of PHL1. We also found that the overexpression of *PHL4* suppresses plant development under both Pi-sufficient and -deficient conditions. Taken together, the results indicate that PHL4 has only a minor role in the regulation of plant responses to Pi starvation and is a negative regulator of plant development.

## Introduction

Phosphorus (P) is an essential nutrient for all organisms. Phosphate (Pi), the major form of P that plants uptake, however, is quite limiting in soil, often resulting in Pi deficiency in natural ecosystems and agricultural lands (Raghothama, 2000; Nussaume et al., 2011). As sessile organisms, plants have evolved sophisticated strategies to cope with this nutritional stress. When grown under Pi starvation conditions, plants trigger a suite of developmental and metabolic responses in order to sustain their growth and development. Some major adaptive responses include the remodeling of root architecture, i.e., the arrest of primary root growth and the enhanced production of lateral roots and root hairs, the increased activity of high-affinity Pi transporters on the root surface, the induction and secretion of acid phosphatases (APases), and the accumulation of anthocyanins and starches in leaves (Yuan and Liu, 2008; López-Arredondo et al., 2014).

**FIGURE 1:**
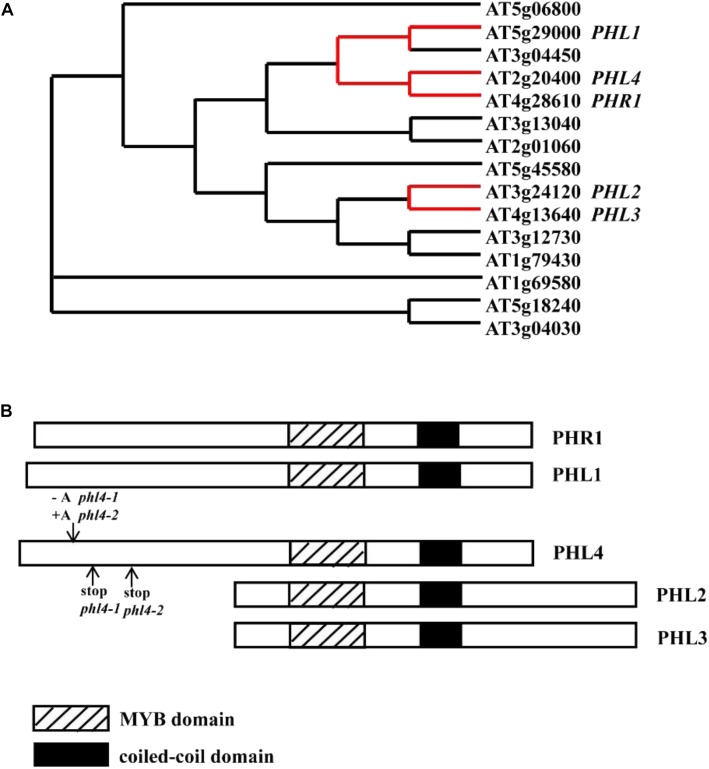
The relative position of PHL4 in the MYB-CC protein family and the mutations in two *phl4* mutant alleles. **(A)** The phylogenetic tree of MYB-CC protein family was generated by an online service^1^. The red lines indicate the positions of *PHR1*, *PHL1*, *PHL2*, *PHL3*, and *PHL4* in the tree. **(B)** Alignment of protein sequences of PHR1, PHL1, PHL2, PHL3, and PHL4. The downward arrow indicated the position where a nucleotide “A” was inserted or deleted due to the gene editing. The upward arrows indicated the positions where the premature stop codon was generated in two mutant alleles. Note that although a similar phylogenetic tree was published by Bustos et al. (2010), we have provided a reconstructed tree to help the reader understand the relationship between PHR1 and PHL1, 2, 3, and 4, and to indicate the positions of the mutation introduced into PHL4 by the CRISPR method.

Numerous transcriptomic studies have demonstrated that underlying these developmental and metabolic changes are changes in the levels of a large number of transcripts which include coding genes, microRNAs, and long non-coding RNAs (Hammond et al., 2003; Wu et al., 2003; Misson et al., 2005; Morcuende et al., 2006; Hernández et al., 2007; Müller et al., 2007; Lundmark et al., 2010; O’Rourke et al., 2013; Secco et al., 2013; Yuan et al., 2016). Therefore, a fundamental question about the molecular mechanism that controls plant responses to Pi starvation is “How are the Pi starvation-induced (PSI) transcriptional changes regulated?” Over the last 20 years, this complicated mechanism has been extensively studied (Puga et al., 2017; Wang et al., 2018). In Arabidopsis, Rubio et al. (2001) identified PHR1 (PHOSPHATE STARVATION RESPONSE 1) as the central regulator of plant transcriptional responses to Pi starvation. PHR1 belongs to a MYB-CC protein family that contains 15 members (Bustos et al., 2010) (**Figure 1A**). The proteins in this family share a MYB domain for DNA-binding and a coiled-coil domain that is involved in protein-protein interactions. PHR1 is a transcription factor, and its mRNA and protein levels are not responsive to changes in Pi availability in the environment (Rubio et al., 2001). PHR1 binds to the *cis*-element called P1BS (PHR1-binding sequence) with an imperfect palindromic sequence GNATATNC, which is prevalent in the promoters of many PSI genes. Knockout of *PHR1* greatly reduces the expression of a number of PSI genes and alters several Pi starvation responses. *PHR1* knockout, for example, decreases the cellular Pi content, decreases anthocyanin accumulation in shoots, and reduces the root-to-shoot ratio (Rubio et al., 2001). In contrast, overexpression of *PHR1* increases the expression of PSI genes and consequently increases the cellular Pi content in plants irrespective of Pi regime (Nilsson et al., 2007). The ability of PHR1 to bind to the P1BS element is further regulated by an SPX domain-containing protein, SPX1, which was proposed to be an intracellular Pi sensor through its binding to inositol pyrophosphates instead of Pi (Puga et al., 2014; Wild et al., 2016). PHR1 also has important roles in the interactions between Pi and other essential nutrients, such as sulfate (Rouached et al., 2011), zinc (Khan et al., 2014), and iron (Bournier et al., 2013). Orthologs of PHR1 have been identified in other plant species, including soybean (Xue et al., 2017), oilseed rape (Ren et al., 2012), rice (Zhou et al., 2008; Lv et al., 2014; Wang et al., 2014b; Guo et al., 2015; Ruan et al., 2017), and wheat (Wang et al., 2013), and they function in a similar manner as PHR1 in plant responses to Pi starvation.

In the Arabidopsis MYB-CC family, PHL1 (PHR1-LIKE 1, At5g29000) is closely related to PHR1 (Bustos et al., 2010). Knockout of *PHL1* did not significantly affect plant transcriptional responses to Pi starvation. In the *phr1phl1* double mutant, however, the induction of about 70% of PSI genes at the transcriptomic level was impaired. Also, the Pi starvation-induced phenotypes that were not observed in *phr1* were observed in the *phr1phl1* double mutant. PHL1 can also bind to the P1BS element and form a heterodimer with PHR1 *in vitro*. These results suggested that PHR1 and PHL1 act redundantly and probably cooperatively to regulate plant responses to Pi starvation.

AtPAP10 (ARABIDOPSIS PURPLE ACID PHOSPHATASE 10) is a major Pi starvation-induced secreted APase (Wang et al., 2011, 2014a; Zhang et al., 2014). Both its transcription and protein accumulation are upregulated by Pi starvation. In our search for the transcription factors that regulate *AtPAP10* expression, we identified two proteins that bind to the minimal functional sequence of the *AtPAP10* promoter (Sun et al., 2016). As shown in **Figure 1A**, these two proteins, designated PHL2 (PHR1-LIKE 2, At3g24120) and PHL3 (At4g13640), are also members of the MYB-CC family. They form a separate clade from PHR1 and PHL1 in the phylogenetic tree. Besides sharing a common MYB domain and a coiled-coil domain, PHR1 and PHL1 contain an extra N-terminal region, and PHL2 and PHL3 contain an extra C-terminal region (**Figure 1B**). RNA-seq analysis of Pi deficient-*phl2* indicated that *PHL2* is another key component controlling plant transcriptional response to Pi starvation (Sun et al., 2016). The structural differences among PHR1, PHL1, PHL2, and PHL3 might be responsible for the subtle differences in their functions, in terms of target genes and transcriptional activities.

In the MYB-CC family, the protein encoded by the gene At2g20400, which we named PHL4 (PHR1-LIKE 4) in this study, shares the highest sequence identity with PHR1. The function of PHL4 in regulating plant responses to Pi starvation, however, has not been characterized due to the lack of the *phl4* mutant. In this work, we generated two *phl4* null mutants using the CRISPR/Cas9 technique. We also generated *phr1phl1* and *phr1phl4* double mutants and *PHL4*-overexpressing lines. Our analyses of these lines indicated that PHL4 has only a minor role in regulating plant responses to Pi starvation and is a negative regulator of plant growth and development.

## Materials and Methods

### Plant Materials and Growth Conditions

All Arabidopsis (*Arabidopsis thaliana*) plants used in this study were in the Colombia-0 ecotype background. The T-DNA insertion lines SALK_067629 (*phr1*) and CS832612 (*phl1*) were obtained from the Arabidopsis Biological Resource Center (ABRC). These two mutants have been proved to be null mutants (Nilsson et al., 2007; Bustos et al., 2010). The *phr1phl1* and *phr1phl4* double mutants were generated through genetic crossing. Seeds were surface-sterilized with 20% bleach for 10 min and washed three times with sterilized ultrapure water. The seeds were then sown on agar or agarose plates containing a Pi-sufficient (+Pi) or Pi-deficient (-Pi) medium. The +Pi medium contained half-strength Murashige and Skoog basal salts (Caisson Labs, catalog no. 01170009), half-strength Murashige and Skoog vitamin powder (1000×) (Phyto Technology Laboratories, catalog no. STT0533013A), 1.0% (w/v) sucrose, 0.5% MES, and 1.2% (w/v) agar (Sigma-Aldrich, catalog no. A1296) or 0.8% (w/v) agarose (Gene Company Ltd., catalog no. 111860). In the -Pi medium, half-strength Murashige and Skoog without Pi replaced the Murashige and Skoog basal salts. All experiments used agar-containing media, except for the experiments concerned with assessment of root development; in the latter case, agarose-containing media were used to aid in the observation of phenotypes. After seeds were stratified for 2 days at 4°C, the agar plates were placed vertically in a growth room with a photoperiod of 16 h light and 8 h dark at 22 to 24°C. The light intensity was 100 μmol m^-2^ s^-1^.

### Generation of the *phl4* Mutants

Two null mutant alleles of *PHL4* were generated using a CRISPR/Cas9-based genome editing system developed by Yan et al. (2015). The targeting sequence in the *PHL4* gene, which is located in the first exon, was determined using an online service^2^. The synthesized DNA fragment containing the targeting sequence of *PHL4* was cloned to the site between two *Bsa* I restriction enzyme sites in the intermediate vector AtU6-26-sgRNA-SK. The fragment between the *Nhe* I and *Spe* I sites of AtU6-26-sgRNA-SK was then further cloned into the *Spe* I restriction enzyme site in the plant vector pCAMBIA1300-pYAO:Cas9. The resultant construct was transformed into Arabidopsis plants of Columbia-0 background. The primary transformants were selected on kaere screened for introduced mutations in *PHL4* by sequencing. The plants with hemizygous mutation in the *PHL4* gene were selfed to produce the homozygous mutants in the next generation. The primers used for construction of gene-editing vectors and verification are listed in **Supplementary Tables 1**, **2**.

### Histochemical Analysis of GUS Activity

Histochemical analysis of GUS activity was carried out as described by Jefferson et al. (1987). After the reactions were completed, the materials were photographed with a camera attached to a stereomicroscope (Olympus SZ61).

### Analysis of Root-Associated APase Activity

Histochemical staining of root-associated APase activity was performed as described by Wang et al. (2011). For quantification of root surface-associated APase activity, two roots were excised from two 7-day-old seedlings, and the roots lengths were measured. The excised roots were thoroughly rinsed with distilled water three times, then transferred to a 2.0-mL Eppendorf tube containing 800 mL of reaction buffer (10 mM MgCl_2_, 50 mM NaAc, 0.1% BCIP, pH 4.9); the tubes with roots were then incubated at 37°C for 1 h before the reaction was terminated by addition of 1 mL of 1M HCl. The samples were centrifuged for 5 min at 10,000 × *g*, and the blue precipitates were re-dissolved in 1M DMSO. Absorbance was measured spectrophotometrically at 635 nm. The root-surface-associated APase activity was expressed as A_635_/cm root length.

### Quantification of Cellular Pi Content and Total P Content

Cellular Pi content and total P content were quantified according to Wang et al. (2011).

### Quantification of Anthocyanin Content

Anthocyanins in shoots were extracted with propanol:HCl:H_2_O (18:1:81, v/v/v) in the dark at room temperature for 24 h. Absorbance was measured at 530 and 650 nm. Anthocyanin content was expressed as (A_530_ – A_650_)/g FW.

### Quantitative Real-Time PCR (qPCR) Analyses of PSI Gene Expression

Total RNAs of 8-day-old seedlings were extracted using the Magen HiPure Plant RNA Mini Kit. A 2-μg quantity of the RNAs was reversely transcribed to cDNA using M-MLV reverse transcriptase (Takara). qPCR analyses were carried out using EvaGreen 2 × qPCR MasterMix (ABM) on a Bio-Rad CFX96 real-time PCR detection system. *ACTIN 2* (At3g18780) mRNA was used as an internal control, and the relative expression level of each gene was calculated by the 2^-ΔΔCt^ method (Livak and Schmittgen, 2001). The primers used for qPCR analysis are listed in **Supplementary Table 3**.

### Vector Construction and Plant Transformation

For analysis of tissue-specific expression patterns of *PHL4*, a 1,403-bp DNA fragment upstream of the start codon was amplified from genomic DNAs by PCR. During the amplification, the DNA sequences overlapping with the restriction enzyme sites of *Xba* I and *Xma* I of the vector were added to the 5′ and 3′ ends of the PCR products. The amplified fragment was cloned into *Xba* I and *Xma* I sites between the *CaMV 35S* promoter and the GUS reporter gene in the plant transformation vector pBI121 using a one-step isothermal *in vitro* recombination procedure (Gibson et al., 2009). For the overexpression of *PHL4*, a 2,735-bp genomic *PHL4* sequence, including both its 5′ and 3′ untranslated regions (UTRs), was cloned into *Bam*H I and *Sac* I restriction enzyme sites after the *CaMV 35S* promoter in the plant transformation vector pZH01 using the same recombination procedure. For analysis of the subcellular localization of PHL4 protein, a 1,194-bp DNA fragment containing the coding sequence (CDS) of *PHL4* was isolated by PCR from plant cDNAs and was cloned into *Kpn* I and *Pst* I restriction enzyme sites between the *CaMV 35S* promoter and GFP gene in the plant transformation vector pJG053, resulting in the construct of *35S::PHL4-GFP*. To generate the *PHL4::PHL4-GFP* construct, the *35S* promoter on the *35S::PHL4-GFP* vector was replaced by the *PHL4* promoter. All constructs were mobilized into *Agrobacterium tumefaciens* strain GV3101 and transformed into Arabidopsis plants via the flower dip method (Clough and Bent, 1998). The primers used for vector construction are listed in **Supplementary Table 1**.

### Luciferase Complementation Imaging (LCI) and Bimolecular Fluorescence Complementation (BiFC) Assays

For LCI assays, the CDSs of *PHL4* and *PHR1* were inserted into the vectors pCAMBIA-nLUC and pCAMBIA-cLUC (Chen et al., 2008), respectively, to generate PHL4-nLUC and cLUC-PHR1 constructs. For BiFC assays, the CDSs of *PHL4* and *PHR1* were individually cloned into the vector nYFP or cYFP (Gibson et al., 2009). The resultant constructs were mobilized into the *A. tumefaciens* strain GV3101 and were used to transform the leaves of *Nicotiana benthamiana*. The LCI and BiFC assays were performed as described by Sun et al. (2016). The primers used for vector construction are listed in **Supplementary Table 4**.

### Electrophoretic Mobility-Shift Assay (EMSA)

For EMSA assays, the full-length CDS of *PHL4* was cloned into the *Not* I and *Sal* I sites after the MBP coding sequence in the vector pMAL-c5x. The CDS of the six times-repeated His tag was added to the C-terminus of the PHL4 protein during the PCR amplification of the CDS of *PHL4*. The resultant construct was transformed into *E. coli* strain BL21 (DE3) for protein production. The MBP-PHL4-His recombinant proteins were purified using Ni-NTA agarose beads. The biotin-labeled probes containing twofold P1BS sequence were generated by annealing the biotin-labeled complementary oligonucleotides. The sequences of the oligonucleotides used for generating the probes are listed in **Supplementary Table 5**. With the purified recombinant proteins and biotin-labeled DNA probes, EMSA was performed as described by Sun et al. (2016).

## Results

### The Expression Patterns of *PHL4*

**FIGURE 2:**
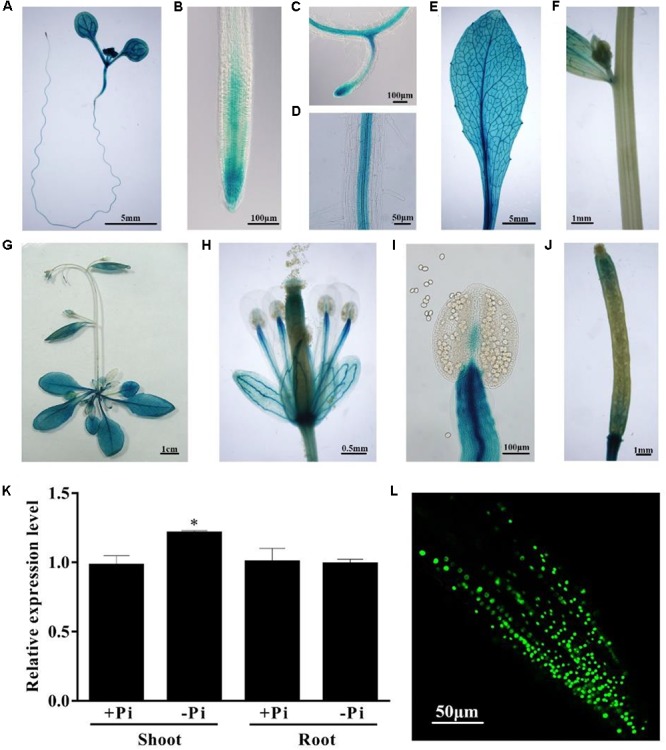
Expression patterns of the *PHL4* gene. **(A–J)** Tissue-specific expression patterns of *PHL4::GUS*. **(A)** An 8-day-old seedling; **(B)** a root tip; **(C)** a newly formed lateral root; **(D)** root mature zone; **(E)** a rosette leaf; **(F)** a stem; **(G)** a 25-day-old mature plant; **(H)** an open flower; **(I)** a stamen; **(J)** a silique. **(K)** Relative expression of the *PHL4* gene in shoots and roots of 8-day-old WT seedlings under +Pi and –Pi conditions as determined by qPCR. Values are the means ± SD of three biological replicates and represent fold changes normalized to transcript levels of the WT on +Pi medium. Means with an asterisk are significantly different from the WT (*P* < 0.05, *t*-test). **(L)** The subcellular localization of the GFP-PHL4 protein in the root tip of a 7-day-old seedling.

To investigate the expression patterns of *PHL4*, we fused a 1,403-bp DNA fragment upstream of its start codon to a GUS reporter gene. This DNA fragment included the 5′ UTR, which contains the first intron, and the 966-bp 5′ flanking sequence. The *PHL4::GUS* construct was transformed into wild-type (WT) Arabidopsis plants. The expression patterns of the GUS gene of a representative line are shown in **Figure 2**. In 8-day-old seedlings, GUS expression was observed in all types of cells of the cotyledon and hypocotyl (**Figure 2A**). GUS expression was detected in the root cap and meristematic zone of both primary and lateral roots but was not detected in the elongation zone of the primary root (**Figures 2B,C**). The GUS gene was strongly expressed in the stele of the maturation zone (**Figure 2D**). In the mature plant, the GUS gene was uniformly expressed in all leaves but was not expressed in the stem (**Figures 2E–G**). In flowers, GUS expression was observed in sepals, filaments, and gynoecia but not in petals or pollen grains (**Figures 2H,I**). In siliques, GUS expression was high at both ends but weak in the middle (**Figure 2J**). Next, we determined whether the expression of *PHL4* was induced by Pi starvation. Both qPCR analysis (**Figure 2K**) and GUS staining assays (**Supplementary Figure 1**) showed that the transcription of *PHL4* was not affected by Pi starvation.

To determine the subcellular localization of the PHL4 protein, we constructed transgenic lines that expressed a PHL4-GFP fusion gene driven by the *35S CaMV* promoter. In the root cells of the transgenic plants, the PHL4-GFP fusion protein was exclusively localized in the nucleus (**Figure 2L**). The localization of PHL4-GFP in the nucleus was confirmed by transient expression assays in the leaves of *N. benthamiana*, in which the expression of *PHL4-GFP* was driven by either the *35S* promoter or the *PHL4* native promoter (**Supplementary Figure 2A**). Using the *35S::GFP-PHL4* transgenic line, we also found that the subcellular localization and protein abundance of PHL4 were not affected by Pi starvation (**Supplementary Figure 2B**).

### Generation of *phl4* Null Mutants

To study the function of *PHL4*, we required *phl4* null mutants for phenotypical characterization. Because the T-DNA knockout mutant of *PHL4* was not available at the Arabidopsis stock centers, we used CRISPR/Cas9 gene editing technique to generate *phl4* mutant alleles. Using this technique, we created four new *PHL4* alleles (*phl4-1* to *phl4-4*, **Supplementary Figure 3**). *phl4-1* had a deletion of one nucleotide ‘A’ at position 130 after the start codon of the *PHL4* gene, which resulted in a frameshift and generated a premature stop codon at position 169. In *phl4-2*, there was an insertion of one nucleotide ‘A’ in the same position as in *phl4-1*, which also caused a frameshift and created a premature stop codon at position 258. *phl4-3* had a 24 nucleotides deletion at position 106, which removed eight amino acids, but did not cause a frameshift. *phl4-4* not only had a six nucleotides deletion at position 127, resulting in the loss of two amino acids, but also had a transition of one nucleotide at two positions. The nucleotide transition from G to A at position 115 caused a non-sense mutation and the nucleotide transition from T to C at position 120 caused a conversion from glutamate to lysine. Because the mutations in *phl4-1* and *phl4-2* would cause a truncation of the PHL4 protein, which result in the elimination of both the MYB domain and the coiled-coil domain (**Figure 1B**), therefore, the proteins encoded by *phl4-1* and *phl4-2* would not be functional, even though the levels of *PHL4* mRNA in these two alleles were not significantly altered compared to that of the WT (**Supplementary Figure 4**). As a consequence, *phl4-1* and *phl4-2* were equivalent to null alleles. Therefore, we chose these two alleles for further studies.

### Responses of the *phl4* Mutants to Pi Starvation

**FIGURE 3:**
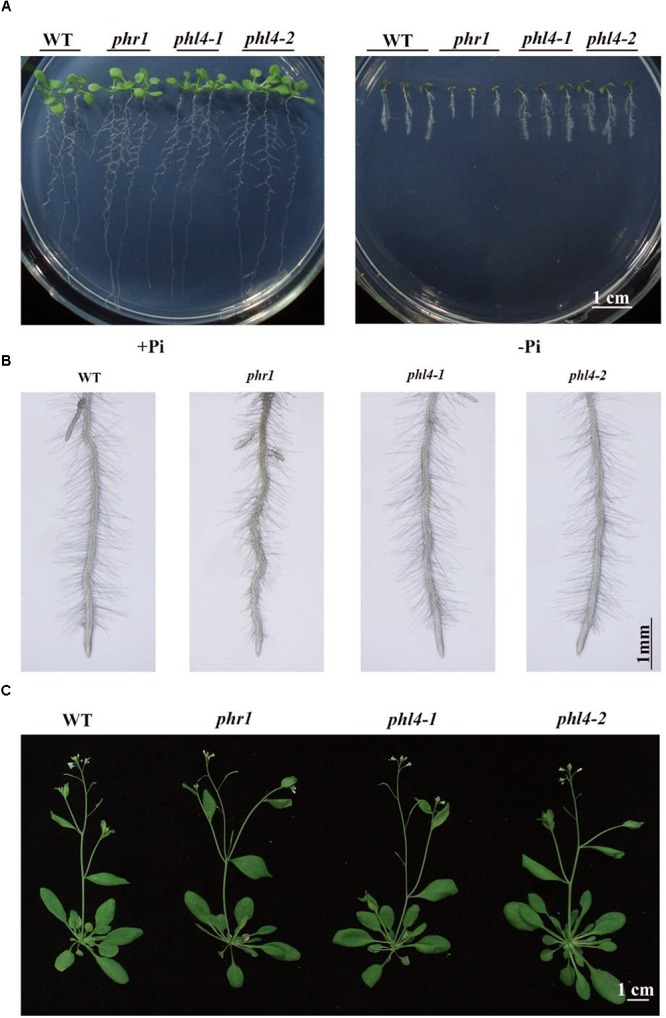
Phenotypic analyses of two *phl4* mutants. **(A)** Morphology of 10-day-old seedlings of the WT, *phr1*, *phl4-1*, and *phl4-2* grown on +Pi and –Pi media. **(B)** Root hairs of 6-day-old seedlings of WT, *phr1*, *phl4-1*, and *phl4-2* grown on –Pi medium. **(C)** Morphology of 5-week-old plants of the WT, *phr1*, *phl4-1*, and *phl4-2* grown in soil.

To determine the roles of *PHL4* in plant responses to Pi starvation, we compared the WT, the *phr1* mutant, and the two *phl4* mutants. The seeds were directly germinated on +Pi and -Pi media. Under Pi sufficiency, the morphologies did not differ among the 10-day-old seedlings (**Figure 3A**). Under Pi deficiency, in contrast, the shoot and root growth of all seedlings were greatly inhibited (**Figure 3A**). The growth inhibition of *phr1*, but not of the two *phl4* mutants, was greater than that of the WT. Enhanced root hair production is a typical response of plants to Pi starvation. The root hair density was similar among the WT, *phr1*, and the two *phl4* mutants; however, *phr1* but not *phl4-1* or *phl4-2* had shorter root hairs than the WT (**Supplementary Figure 5**). No obvious morphological difference was observed among these plants after 5 weeks of growth in soil (**Figure 3C**).

**FIGURE 4:**
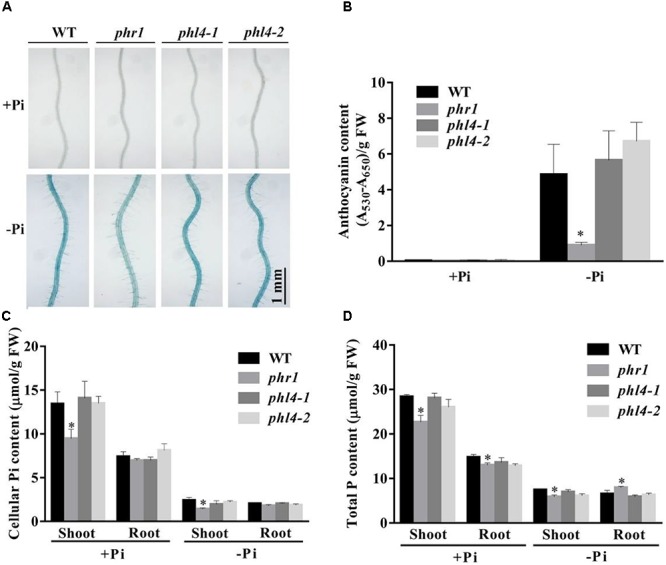
Analyses of root-associated APase activity and contents of anthocyanin, cellular Pi, and total P in the WT, *phr1*, *phl4-1*, and *phl4-2* on +Pi and –Pi media. **(A)** The APase activities revealed by BCIP staining of the root surfaces of 6-day-old seedlings of the WT and various mutants. **(B)** Anthocyanins in the shoots of 12-day-old seedlings of the WT and various mutants. **(C,D)** Cellular Pi and total P contents in the shoots and roots of 12-day-old seedlings of the WT and various mutants. These experiments were repeated three times with similar results. Values represent means ± SD of three replicates. Means with asterisks are significantly different from the WT (*P* < 0.05, *t*-test).

Two additional hallmark responses of plants to Pi starvation are induction and secretion of APases on the root surface and the accumulation of anthocyanins in shoots. We analyzed these two traits for the *phl4* mutants. Root surface-associated APase activity can be detected by histochemical staining using a substrate of APase, BCIP (5-bromo-4-chloro-3-indolyl phosphate). The product of the enzyme reaction forms a blue precipitate. Under Pi deficiency and compared to the WT, secreted APase activity as indicated by BCIP staining was substantially reduced in *phr1* but not in the two *phl4* mutants (**Figure 4A**). This conclusion was further supported by the quantitative analysis of secreted APase activity of these seedlings (**Supplementary Figure 6**). Accumulation of anthocyanins in shoots was also significantly reduced in *phr1* but not in the two *phl4* mutants (**Figure 4B**). Next, we examined the cellular Pi and total P contents in these plants. When the plants were grown on either +Pi or -Pi media, the cellular Pi content of shoots was reduced in *phr1* but not in the two *phl4* mutants relative to the WT (**Figure 4C**). The Pi contents of roots did not significantly differ among the WT, the *phr1* mutant, and the two *phl4* mutants (**Figure 4C**). Although the total P content in shoots was about 15% lower for *phr1* than for the WT under both Pi sufficiency and deficiency (**Figure 4D**), the total P content in roots was lower for *phr1* than for the WT under Pi sufficiency but was higher for *phr1* than for the WT under Pi deficiency (**Figure 4D**). The total P content of roots did not differ between the two *phl4* mutants and the WT under Pi sufficiency or deficiency.

**FIGURE 5:**
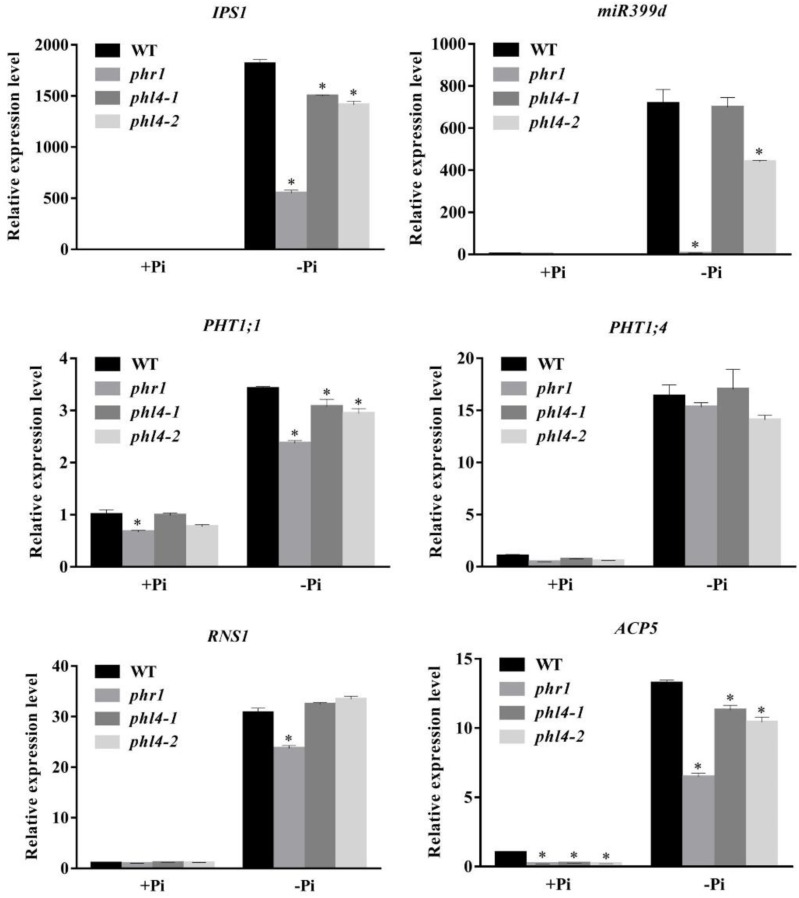
Analyses of PSI gene expression in the WT, *phr1*, *phl4-1*, and *phl4-2*. Roots of 8-day-old seedlings of the WT and other mutants grown on +Pi and –Pi media were used for qPCR analysis. The names of the genes examined are indicated on the top of each panel. Values are the means ± SD of three biological replicates and represent fold changes normalized to transcript levels of the WT on +Pi medium. Means with asterisks are significantly different from the WT (*P* < 0.05, *t*-test).

We then analyzed the effect of the *PHL4* mutation on the expression of six PSI marker genes. These PSI genes included a non-coding transcript, *IPS1* (Burleigh and Harrison, 1999); a microRNA, *miR399d* (Fujii et al., 2005); two high-affinity phosphate transporters, *AtPT1* (*Pht1;1*) and *AtPT2* (*Phtl;4*) (Muchhal et al., 1996); a ribonuclease, *RNS1* (Bariola et al., 1994); and an acid phosphatase, *ACP5* (*AtPAP17*) (del Pozo et al., 1999). The induction of these six PSI genes, and especially of *IPS1* and *miRNA399d*, was substantially lower in *phr1* roots than in WT roots (**Figure 5**). In the two *phl4* mutants, however, the induction of expression was unaffected for two of the six genes and was only mildly reduced for four of the six genes.

Taken together, these results indicate that the mutation of the *PHL4* gene had only a minor effect on plant transcriptional responses to Pi starvation. As a consequence, the mutation of the *PHL4* gene did not significantly alter the physiology or development of plants under Pi starvation.

### Functional Analysis of the *phr1phl4* Double Mutant

**FIGURE 6:**
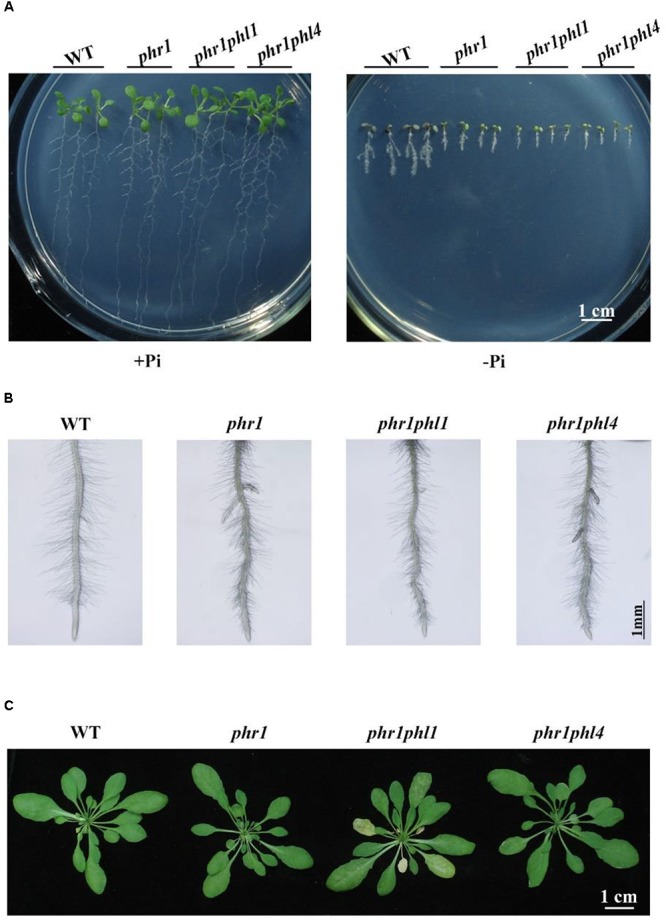
Phenotypic analysis of *phr1phl1* and *phr1phl4* double mutants. **(A)** Morphology of 10-day-old seedlings of the WT, *phr1*, *phr1phl1*, and *phr1phl4* grown on +Pi and –Pi media. **(B)** Root hairs of 6-day-old seedlings of WT, *phr1*, *phr1phl1*, and *phr1phl4* grown on –Pi medium. **(C)** Morphology of 5-week-old plants of the WT, *phr1*, *phr1phl1*, and *phr1phl4* grown in soil. The flower stalks were removed.

Because the mutation of *PHL4* did not cause obvious phenotypical changes, we wondered whether there was a functional redundancy among the members of the MYB-CC family. To investigate this possibility, we generated a *phr1phl4* double mutant by crossing *phr1* (SALK_067629) with *phl4-2*. The primers used are listed in **Supplementary Table 2**. PHL1 is another close homolog of PHR1 (**Figure 1A**). The mutation of *PHL1* alone does not affect PSI gene expression or cause any other obvious phenotypical changes (Bustos et al., 2010). The combining of the *phr1* and *phl1* mutations, however, has a synergistic effect on the plant transcriptional responses to Pi deficiency, as well as on other traits, such as root hair development (Bustos et al., 2010). To assess the combined effects of *PHL4* and *PHR1* mutations, we compared the growth phenotypes and plant responses to Pi starvation between *phr1phl1* and *phr1phl4*. When grown on +Pi medium for 10 days, the WT, *phr1*, *phr1phl1*, and *phr1phl4* did not display obvious morphological differences. On -Pi medium, the overall growth was more inhibited for *phr1* than for the WT, and the growth of *phr1phl1* and *phr1phl4* double mutants was more inhibited than that of the *phr1* single mutant. These results indicated a synergistic effect of the double mutation and a similar effect of *PHL1* and *PHL4* mutations on *phr1* (**Figure 6A**). As shown in **Figure 6B**, the WT, *phr1*, and the two double mutants had similar root hair density. Compared to *phr1*, root hair length of *phr1phl1* was further reduced, but such a reduction was not evident for *phr1phl4* (**Figure 6B** and **Supplementary Figure 5**). When grown in soil for 5 weeks, *phr1* and the WT had a similar growth phenotype whereas *phr1phl1* showed a sign of early senescence (**Figure 6C**), which was consistent with that reported by Bustos et al. (2010). The growth phenotype of *phr1phl4* was also similar to that of the WT and *phr1*.

**FIGURE 7:**
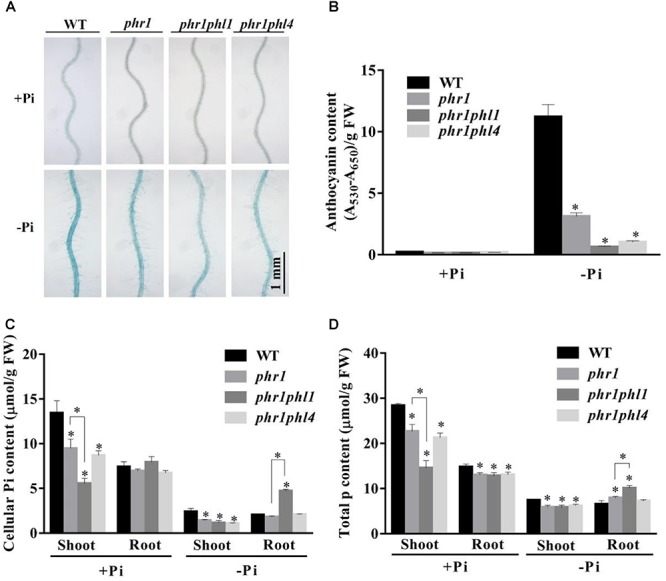
Analyses of root-associated APase activity, anthocyanin, cellular Pi, and total P contents of the WT, *phr1*, *phr1phl1*, and *phr1phl4* on +Pi and –Pi media. **(A)** The APase activities revealed by BCIP staining on the root surfaces of 6-day-old seedlings of the WT and various mutants. **(B)** Anthocyanins in the shoots of 12-day-old seedlings of the WT and various mutants. **(C,D)** Cellular Pi and total P contents in the shoots and roots of 12-day-old seedlings of the WT and various mutants. These experiments were repeated three times with similar results. Values represent means ± SD of three replicates. Means with asterisks are significantly different from the WT (*P* < 0.05, *t*-test). Means with asterisks and thin lines are significant different from each other (*P* < 0.05, *t*-test).

Next, we examined the root-associated APase activity and anthocyanin accumulation in *phr1phl4*. The root-associated APase activity and anthocyanin accumulation in shoots were lower in both *phr1phl1* and *phr1phl4* than in *phr1* but did not significantly differ between *phr1phl1* and *phr1phl4* (**Figures 7A,B**). This conclusion was also supported by the quantitative analyses of the APase activity of these plants (**Supplementary Figure 6**). In terms of cellular Pi and total P contents, the mutation of *PHL1* and *PHL4* had different effects on the *phr1* mutant. Under Pi sufficiency, cellular Pi and total P contents in shoots were lower in *phr1phl1* than in *phr1*; under Pi deficiency, however, cellular Pi and total P contents in shoots were similar in *phr1phl1* and *phr1* (**Figures 7C,D**). In the shoots of *phr1phl4*, however, the levels of cellular Pi and total P were similar to those of *phr1* under both Pi sufficiency and Pi deficiency. In Pi-sufficient roots, levels of cellular Pi and total P in *phr1phl1* and *phr1phl4* were similar to those in *phr1*. In Pi-deficient roots, however, levels of cellular Pi and total P were significantly higher in *phr1phl1* than in the WT or *phr1*. Cellular Pi and total P contents did not significantly differ between *phr1phl4* and *phr1*.

**FIGURE 8:**
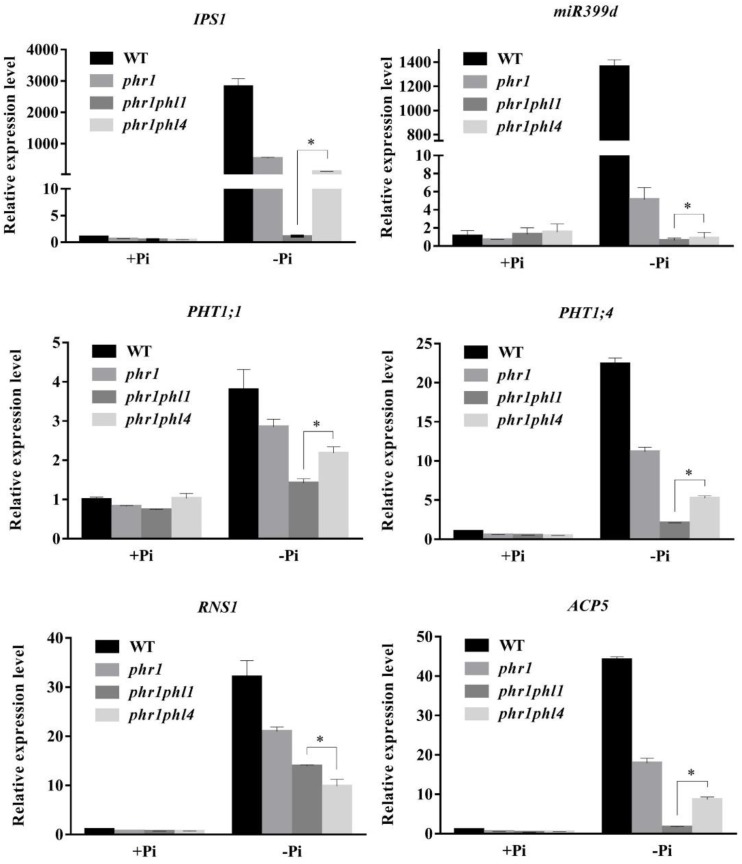
Analysis of PSI gene expression in the WT, *phr1*, *phr1phl1*, and *phr1phl4*. Roots of 8-day-old seedlings of the WT and other mutants grown on +Pi and –Pi media were used for qPCR analysis. The names of the genes examined are indicated on the top of each panel. Values are the means ± SD of three biological replicates and represent fold changes normalized to transcript levels of the WT on +Pi medium. Means with asterisks and thin lines are significantly different from each other (*P* < 0.05, *t*-test).

We then compared the expression of the six PSI genes in the WT and the three mutants. The induction of all six PSI genes was significantly reduced in *phr1* and was further reduced in *phr1phl1* (**Figure 8**). Although the induction of PSI genes was lower in *phr1phl4* than in *phr1*, the reduction was less in *phr1phl4* than in *phr1phl1*.

These results suggest that, like *PHL1*, *PHL4* acts redundantly with *PHR1* in plant responses to Pi deficiency. The contribution to the response, however, might be less for *PHL4* than for *PHL1*.

### Overexpression of *PHL4* Alters Plant Growth and Development

**FIGURE 9:**
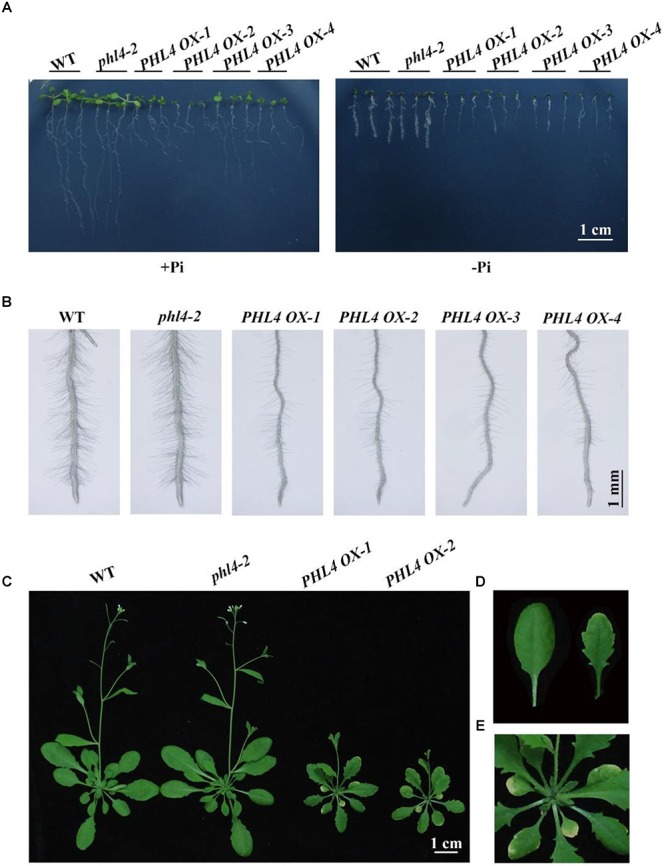
Phenotypic analyses of the *PHL4*-overexpression lines. **(A)** Morphology of 8-day-old seedlings of the WT, *phl4*, and four *PHL4*-overexpression lines grown on +Pi and –Pi media. **(B)** Root hairs of 6-day-old seedlings of the WT, *phl4*, and four *PHL4*-overexpression lines grown on –Pi medium. **(C)** Morphology of 5-week-old seedling of the WT, *phl4*, and two *PHL4*-overexpression lines grown in soil. **(D)** A comparison of the leaf edge of the WT and *PHL4 OX-1*. **(E)** A close view of the rosette of *PHL4 OX-1*.

Another way to overcome genetic redundancy in the functional characterization of a given gene is to analyze the phenotypes of its overexpressing lines (Weigel et al., 2000). We therefore generated *PHL4*-overexpressing lines (*PHL4 OX*) by transforming *phl4-2* with a genomic sequence of the WT *PHL4* gene driven by a *35S CaMV* promoter. More than 10 independent transgenic lines were generated, and the phenotypes of some representative lines were analyzed. Under both Pi sufficiency and deficiency, *phl4* showed similar growth phenotypes as the WT, while the overall growth of four transgenic lines was greatly impaired (**Figure 9A**). On +Pi medium, the 8-day-old seedlings of these four overexpressing lines had small, epinastic cotyledons and short primary roots with abnormal gravitropism; on -Pi medium, the *PHL4-OX* seedlings were once again smaller than WT and *phl4* seedlings, but the abnormal gravitropism was no longer evident. Root hair density and root hair length were also significantly lower for these four transgenic lines than for the WT and *phl4* (**Figure 9B** and **Supplementary Figure 5**). When the *PHL4*-overexpressing lines were grown in soil for 5 weeks, they were small with curled and serrated rosette leaves, and had early senescence (**Figures 9C–E**).

**FIGURE 10:**
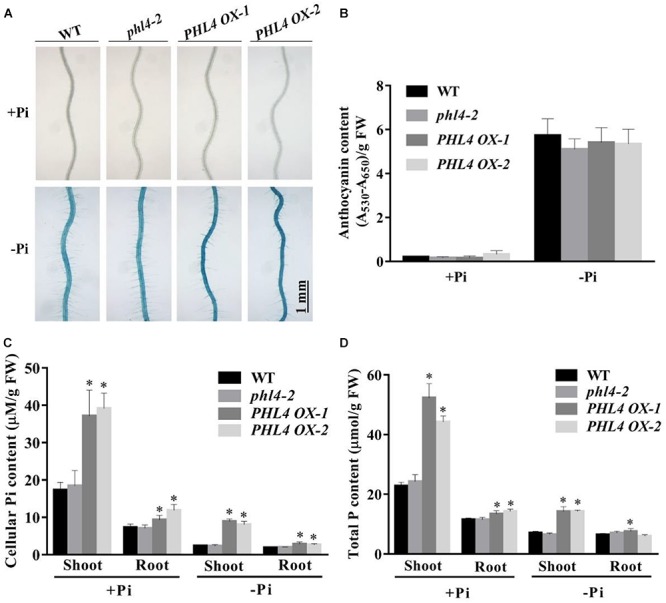
Analyses of root-associated APase activity, anthocyanin, cellular Pi, and total P contents of the two *PHL4*-overexpressing lines (*PHL4 OX-1* and *PHL4 OX-2*) grown on +Pi and –Pi media. **(A)** The APase activities revealed by BCIP staining on the root surfaces of 6-day-old seedlings of the WT, *phl4*, and *PHL4 OX-1* and *-2*. **(B)** Anthocyanins in the shoots of 12-day-old seedlings of the WT, *phl4*, and *PHL4 OX-1* and *-2*. **(C,D)** Cellular Pi and total P contents in the shoots and roots of 12-day-old seedlings of WT, *phl4*, and *PHL4 OX-1* and *-2*. These experiments were repeated three times with similar results. Values represent means ± SD of three replicates. Means with asterisks are significantly different from the WT (*P* < 0.05, *t*-test).

**FIGURE 11:**
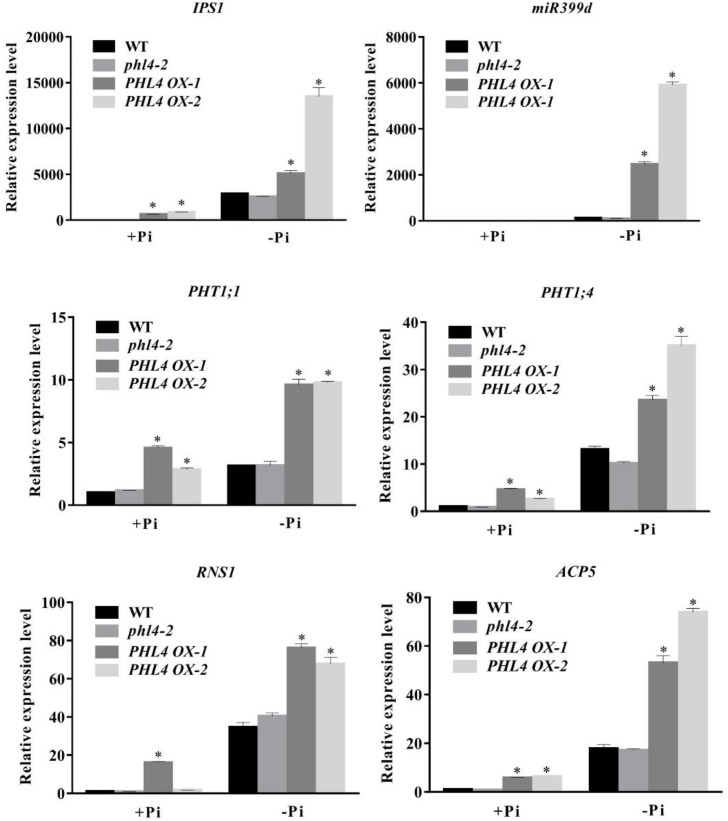
Analyses of PSI gene expression in two *PHL4*-overexpressing lines (*PHL4 OX-1* and *PHL4 OX-2*). Roots of 8-day-old seedlings of the WT, *phl4*, and *PHL4 OX-1* and *-2* grown on +Pi and –Pi media were used for qPCR analysis. The names of the genes examined are indicated on the top of each panel. Values are the means ± SD of three biological replicates and represent fold changes normalized to transcript levels of the WT on +Pi medium. Means with asterisks are significantly different from the WT (*P* < 0.05, *t*-test).

In response to Pi starvation, root-associated APase activity was greater in the *PHL4 OX* lines than in the WT or *phl4* as revealed by BCIP staining (**Figure 10A**); however, the quantitative analysis using BCIP as the substrate showed that the total root-associated APase activity per cm root length was similar between the WT and *PHL4 OX* lines (**Supplementary Figure 6**). This was probably due to that the *PHL4 OX* line had less and shorter root hairs and thinner primary root, even though they had stronger BCIP than the WT on their root surface. The anthocyanin contents in shoots were similar in the *PHL4 OX* lines, the WT, and *phl4* (**Figure 10B**). In shoots, the cellular Pi and total P contents were two times greater in two of the overexpressing lines than in the WT or *phl4* grown on either +Pi or -Pi media (**Figures 10C,D**). In roots, the *PHL4 OX* lines also had increased cellular Pi and total P contents, although the increase was less than in the shoots. In response to Pi starvation, the induction of the six typical PSI genes was much higher in the two *PHL4 OX* lines than in the WT or *phl4* (**Figure 11**).

### Physical Interaction Between PHL4 and PHR1

**FIGURE 12:**
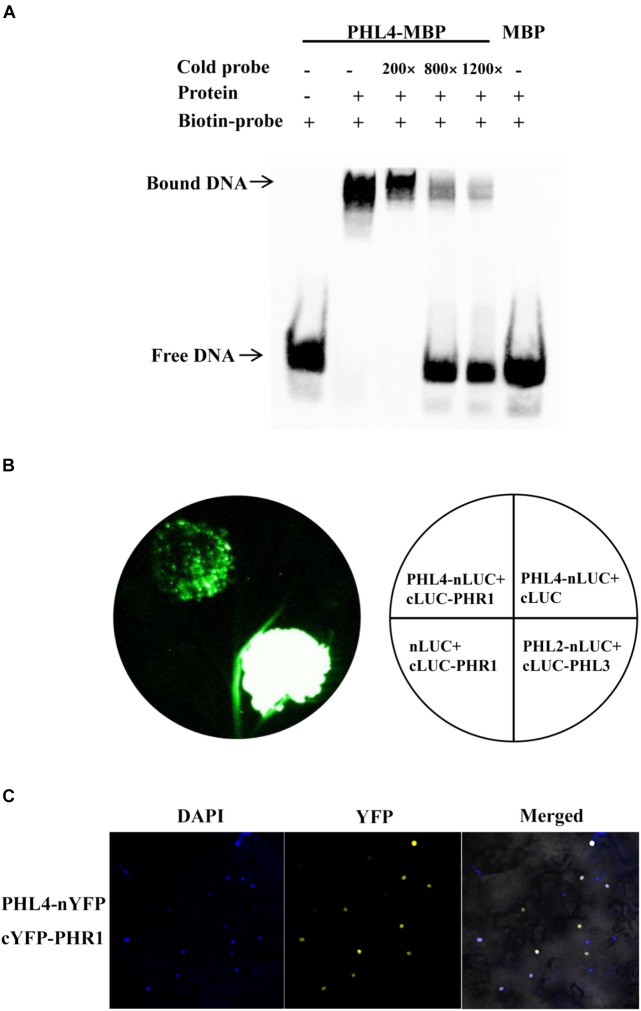
PHL4 binds to the P1BS element and interacts with PHR1. **(A)** EMSA assay showing that PHL4 protein specifically binds to the P1BS element. The experiment was performed using 4 μg of MBP-PHL4 protein and 10 fmol of biotin-labeled DNA probe. The same unlabeled probe was used as the competitor at an excess molar ratio of 200:1, 800:1, and 1200:1 to labeled probe. **(B)** LCI assays for the interaction of PHL4 and PHR1. The constructs PHL4-nLUC and cLUC-PHR1 were co-infiltrated into the leaves of *N. benthamiana*, and LUC signals were recorded 2 d after infiltration. **(C)** BiFC assays for the interaction between PHL4 and PHR1. The constructs PHL4-nYFP and cYFP-PHR1 were co-infiltrated into the leaves of *N. benthamiana*. YFP fluorescence was detected 2 days after infiltration by confocal microscopy. The nuclei were revealed by DAPI staining.

Because PHL4 shares the highest sequence homology with PHR1, and because knockout or overexpression of *PHL4* altered the expression of PSI genes, we wondered whether *PHL4* could also bind to the P1BS element in order to regulate gene expression. A MBP (maltose-binding protein)-PHL4-His recombinant protein was produced in *E. coli*, and the fusion protein was purified to homogeneity with an His affinity column. An EMSA demonstrated that PHL4-MBP, but not MBP alone, could form a complex with the biotin-labeled DNA probe containing the P1BS sequence (**Figure 12A**). An excess of non-labeled DNA probe competed with the labeled DNA for the binding with PHL4-MBP, indicating that such binding was sequence-specific. We next used a luciferase complementary imaging (LCI) assay to determine whether PHL4 can directly interact with PHR1. PHL4 was fused with the N-terminal half of a luciferase (LUC) (PHL4-nLUC), and PHR1 was fused with the C-terminal half of LUC (cLUC-PHR1). These two constructs were co-transformed into the leaves of *N. benthamiana* with proper controls. As positive controls, PHL2 and PHL3 showed a strong interaction (**Figure 12B**), which was previously reported (Sun et al., 2016). In contrast, no reconstituted LUC signals were detected for the pairs of PHL4-nLUC and cLUC or nLUC and cLUC-PHR1. In the leaves in which PHL4-nLUC and cLUC-PHR1 were co-expressed, a moderately strong signal of reconstituted LUC was detected, indicating that PHL4 can interact with PHR1. The interaction between PHL4 and PHR1 was further confirmed by a bimolecular fluorescence complementation (BiFC) assay in the leaves of *N. benthamiana*, and the results showed that the interaction occurred in the nucleus (**Figure 12C**).

## Discussion

Plants use many responses to cope with Pi deficiency in the environment. These adaptive responses are controlled by complicated regulatory networks at transcriptional (Bustos et al., 2010), posttranscriptional (Fujii et al., 2005; Bari et al., 2006; Franco-Zorrilla et al., 2007), and posttranslational (Liu et al., 2012; Lin et al., 2013) levels. Among these regulatory processes, a crucial role of transcriptional regulation has been well established by transcriptomic studies (Jain et al., 2012). The functional characterization of the Arabidopsis transcription factor PHR1 and its homologs within the MYB-CC protein family, PHL1 and PHL2, revealed the framework of a central regulatory system controlling plant transcriptional responses to Pi starvation. In the double knockout mutants *phr1phl1* and *phr1phl2*, however, the expression of PSI genes was not completely abolished (Bustos et al., 2010; Sun et al., 2016), suggesting that other transcription factors in or out of the MYB-CC family also contribute to the regulation of plant transcriptional responses to Pi starvation. In the Arabidopsis MYB-CC protein family, although the PHR1 homologs PHL1, PHL2, and PHL3 have been shown to regulate Pi responses, the functions of the most closely related homolog of PHR1, which we named PHL4 (At2g20400) (**Figure 1A**), had not been previously studied due to the lack of its knockout mutant.

As a first step toward understanding the functions of PHL4 in plant responses to Pi starvation and in plant development, we analyzed the expression patterns of *PHL4* as affected by Pi availability. Our results indicated that PHL4 is localized in the nucleus and that neither its transcription nor its protein abundance is affected by external Pi levels (**Figures 2K,L**). These features are similar to those of PHR1.

To determine the roles of PHL4 in plant responses to Pi starvation, we generated two *phl4* mutant alleles using the CRISPR/Cas9 gene editing technique. The premature stop codons introduced into the *PHL4* gene through gene editing eliminated both the MYB domain and the coiled-coil domain; therefore, the two resultant mutants could be regarded as null alleles (**Figure 1B**). We found that the functional disruption of *PHL4* reduced the expression of only four of the six PSI marker genes examined, and that the reduction was much lower than that observed for the *phr1* mutant (**Figure 5**). Furthermore, although the mutation of *PHL4* somewhat reduced the expression of four PSI marker genes, it did not cause obvious alterations in the major hallmark responses to Pi starvation, i.e., mutation of *PHL4* did not affect root architecture (**Figure 3**), root-associated APase activity, the accumulation of anthocyanins in shoots, or the cellular Pi and total P contents in shoot and roots (**Figure 4**). These results suggest that PHL4 has only a minor role in regulating plant responses to Pi starvation.

The mild effect of *PHL4* mutation on plant responses to Pi starvation might be due to the functional redundancy among members of the MYB-CC family. To test this possibility, we generated the *phr1phl4* double mutant and analyzed its phenotypes along with those of the *phr1* single mutant and the *phr1phl1* double mutant. Three different types of effects of *phl1* and *phl4* mutations on *phr1* were observed: First, the *phl1* and *phl4* mutations had similar enhancing effects on certain *phr1* phenotypes. Under Pi deficiency, for example, the growth of *phr1* was more inhibited than that of the WT, and this inhibitory effect was further enhanced and to a similar degree in both *phr1phl1* and *phr1phl4* (**Figure 6A**). Such similar enhancing effects were also observed in *phr1phl1* and *phr1phl4* for the induction of root-associated APase activity (**Figure 7A**) and for the accumulation of anthocyanins in shoots (**Figure 7B**). Second, the enhancing effect was greater for *phl1* than for *phl4* with regard to the expression of six PSI marker genes (**Figure 8**). Third, the *phl1* mutation but not the *phl4* mutation enhanced the following *phr1* phenotypes: root hair production by seedlings grown on -Pi medium (**Figure 6B**), early senescence phenotypes of plants grown in soil (**Figure 6C**), and Pi and total P contents in +Pi shoots and -Pi roots (**Figures 7C,D**). Based on these results, we conclude that PHL4 has a smaller role than PHL1 in regulating Pi starvation responses, although PHL4 is more closely related to PHR1 than PHL1.

When analyzing the cellular Pi and total P contents in shoots and roots, we found that the patterns for cellular Pi content were quite similar to those for total P content, indicating that the amount of free Pi in the plant tissues was correlated with the total P content, which includes both free Pi and P incorporated into organophosphates, such as nucleic acids and phospholipids. Rubio et al. (2001) and Bustos et al. (2010) previously noticed that cellular Pi content was decreased in the whole seedlings of *phr1*, and was further decreased in the whole seedlings of the *phr1phl1* double mutant. Nilsson et al. (2007) observed that under Pi deficiency, Pi content but not total P in *phr1* was decreased in shoots and that both Pi and total P contents were increased by about two times in roots, suggesting that PHR1 is involved in Pi translocation from roots to shoots. Under Pi deficiency in our experiments, both Pi and total P contents were decreased in *phr1* shoots, and total P but not cellular Pi was slightly increased in *phr1* roots (**Figures 7C,D**). This discrepancy between Nilsson et al. (2007) and the current research may be due to the use of hydroponics in their study vs. agar plates in our study. However, we also documented an increase of both Pi and total P contents in the roots of the *phr1phl1* double mutant under Pi deficiency, but not in the roots of *phr1phl4* under Pi deficiency (**Figures 7C,D**). Based on these results, we propose that PHR1 and PHL1 together, but not PHR1 and PHL4 together, function in the regulation of Pi translocation from roots to shoots. PHO1 was previously identified as a key component in regulating Pi translocation from roots to shoots (Poirier et al., 1991). However, whether the over-accumulation of Pi in roots of *phr1phl1* grown under Pi deficiency is linked to PHO1 function requires further investigation.

Another way to overcome genetic redundancy when investigating the functions of a given gene is to study the phenotypes of its overexpressing lines (Weigel et al., 2000). We therefore generated *PHL4 OX* lines using a *35S CaMV* promoter in the *phl4* background. When grown on +Pi medium, the seedlings of four *PHL4 OX* lines had short primary roots that lacked gravitropism and that had small cotyledons with epinasty (**Figure 9A**). On -Pi medium, the *PHL4 OX* seedlings were smaller than the WT, but they regained gravitropism. Under Pi deficiency, root hair development was greatly inhibited in the overexpressing lines (**Figure 9B**). Although *PHL4* is not expressed in root hairs of the WT (**Figure 2D**), *PHL4* might be ectopically overexpressed in trichoblasts of the overexpressing lines, and such ectopic expression may have inhibited root hair initiation and elongation. When grown in soil for 5 weeks, *PHL4 OX* plants were much smaller than WT or *phl4* plants and had yellowish and serrated leaves (**Figure 9C**). The inhibitory effects of *PHL4* overexpression on both shoot and root growth suggest that PHL4 is also a negative regulator of plant growth.

Regarding other plant responses to Pi starvation, the overexpression of *PHL4* enhanced root-associated APase activity (**Figure 10A**), increased tissue Pi and total P contents, especially in shoots (**Figures 10C,D**), and enhanced the expression of all PSI marker genes examined, although the degree of enhancement varied from line to line due to the position effect (**Figure 11**). This enhancement of gene expression caused by the overexpression of *PHL4* is similar to that caused by overexpression of *PHR1* (Nilsson et al., 2007; Sun et al., 2016). However, the enhancement of anthocyanin accumulation in shoots was not observed in the overexpressing lines grown on -Pi medium (**Figure 10B**). When *PHR1* was overexpressed, in contrast, anthocyanin accumulation was enhanced under Pi deficiency. These results suggest that overexpression of *PHL4* affects most but not all plant responses to Pi starvation.

If PHL4 is indeed a transcription factor, it should be able to bind to DNA molecules. PHR1, PHL1, PHL2, and PHL3 were previously demonstrated to bind to the P1BS element (Rubio et al., 2001; Bustos et al., 2010; Sun et al., 2016). Nguyen et al. (2016) studied the regulation of a pollen-specific phospholipase gene, PLA_2_-γ. They found that PHL4, which they named γMYB1, and another MYB-CC family member (At3g13040), which they named γMYB2, could bind to the P1BS element on the promoter of the phospholipase gene, PLA2-γ. Our EMSA experiments corroborated that PHL4 can bind to the P1BS element in a sequence-specific manner (**Figure 12A**). Nguyen et al. (2016) also showed that γMYB1 (PHL4) but not γMYB2 has the activity of a transcriptional activator. This is consistent with our finding that the overexpression of *PHL4* enhanced the expression of six PSI genes, all of which carried the P1BS element. PHL1 has been previously shown to form a heterodimer with PHR1 *in vitro* (Bustos et al., 2010). Because PHL4 is most closely related to PHR1, we therefore wondered whether PHL4 could also directly interact with PHR1. Indeed, our LCI and BiFC assays demonstrated that PHL4 could directly interact with PHR1 in the nucleus (**Figures 12B,C**). Because both PHL1 and PHL4 can form a protein complex with PHR1, knockout of either one may be compensated for by the other. This might explain why the *phl1* and *phl4* single mutants did not display an obvious phenotype in plant responses to Pi starvation.

In summary, our analyses of two *phl4* null mutants indicated that PHL4, like PHL1, acts redundantly with PHR1 to regulate plant responses to Pi starvation; however, its role in the PHR1-mediated central regulatory system is only minor. Our comparative study of *phr1phl1* and *phr1phl4* double mutants revealed both overlapping and distinct functions of PHL1 and PHL4 in regulating plant responses to Pi starvation and suggested that the contribution of PHL4 to such regulatory system is less than that of PHL1. Moreover, the phenotypes of *PHL4 OX* lines suggested that PHL4 is a negative regulator of plant growth and development. The next challenge will be to understand how PHL1 and PHL4 differentially act with PHR1 to regulate plant responses to Pi starvation.

## Author Contributions

ZW, ZZ, and DL conceived and designed the experiments. ZW and LS carried out the experiments. ZW and DL analyzed the data. ZW and DL wrote the manuscript.

## Conflict of Interest Statement

The authors declare that the research was conducted in the absence of any commercial or financial relationships that could be construed as a potential conflict of interest.

## Footnotes

1 http://www.ebi.ac.uk/Tools/msa/clustalo/

2 http://www.genome.arizona.edu/crispr/CRISPRsearch.html
