# Supplementary material for: Functional Characterization of Arabidopsis PHL4 in Plant Response to Phosphate Starvation
Source: Front Plant Sci. 2018 Oct 1;9:1432. doi: 10.3389/fpls.2018.01432 (PMC6174329; doi:10.3389/fpls.2018.01432)
Supplement: Supplementary file 1 [file Table_1.DOCX]

**Supplementary Table 1**. Primers used for construction of plant transformation vectors

| **Primer name** | **Primer sequence** |
| --- | --- |
| PHL4-CRISPR-F | 5’-ATTGTCCGTCGGAATTGACCTTAA-3’ |
| PHL4-CRISPR-R | 5’-AAACTTAAGGTCAATTCCGACGGA-3’ |
| pJG053-PHL4-F-KpnI | 5’-ACGCGTCCCGGGGCGGTACC ATGATCCCGAATGATGATGATG-3’ |
| pJG053-PHL4--R-PstI | 5’-TACGAACGAAAGCTCTGCAG TCATTCTTCATTTCTCGGACGC-3’ |
| PBI121-PHL4-F-XbaI | 5’-GCCTGCAGGTCGACTCTAGA GTTTTGCAACTTCTTATTTAG-3’ |
| PBI121-PHL4-R-XmaI | 5’-AGGGACTGACCGTACCCGGG CACTCGAGTTTCTGAATTACTC-3’ |
| PZH01-PHL4-F-BamHI | 5’-CGGGGGACTCTAGAGGGATCC AGAGAACAAAGCGTGAGAGAG-3’ |
| PZH01-PHL4-R-SacI | 5’-GATCGGGGAAATTCGAGCTC CATGAGTTGATTCCATACATGC-3’ |

**Supplementary Table 2.** Primers used for double mutant identification

| **Primer name** | **Primer sequence** |
| --- | --- |
| PHL1-LP | 5’-GTGGAGACGTTTCTGCACTTC-3’ |
| PHL1-RP | 5’-TCCCACAATCCAAATTCAGAG-3’ |
| PHR1-LP | 5’-TGCATTAGCAGGGAACTAAAGAA-3’ |
| PHR1-RP | 5’-GACCATTAGGACAAACCTACCA-3’ |
| LBb1.3 | 5’-ATTTTGCCGATTTCGGAAC-3’ |
| PHL4-crispr-F1 | 5’-ATTGGCGTTAGGTTTGGTGAT-3’ |
| PHL4-crispr-R | 5’-TTGCATACTTGGTTGCTGGAC-3’ |

**Supplementary Table 3.** Primers used for qPCR analyses of PSI gene expression

| **Primer name** | **Primer sequence** |
| --- | --- |
| ACT2-F | 5’-GACCTTGCTGGACGTGACCTTAC-3’ |
| ACT2-R | 5’-GTAGTCAACAGCAACAAAGGAGAGC-3’ |
| IPS1-F | 5’-AGACTGCAGAAGGCTGATTCAGA-3’ |
| IPS1-R | 5’-TTGCCCAATTTCTAGAGGGAGA-3’ |
| PHT1;1-F | 5’-TGATGATCTTGTGCTCTGTCG-3’ |
| PHT1;1-R | 5’-ATGACACCCTTGGCTTCGT-3’ |
| PHT1;4-F | 5’-CGAAGCTCCTCGGTCGTAT-3’ |
| PHT1;4-R | 5’-GGAGAGTCCCAGGCTTTTGT-3’ |
| RNS1-F | 5’-TTGTTATCCAAATTCAGGCAAA-3’ |
| RNS1-R | 5’-AGTTAGGCCAAAGACCATGAAT-3’ |
| miR399-F | 5’-AATACTCCTATGGCAGATCGCATTGG-3’ |
| miR399-R | 5’-TCCTTTGGCAGAGAAGCATTTTACTTG-3’ |
| ACP5-F | 5’-CTTAAGTCCTATTGCAGGCTAGGT-3’ |
| ACP5-R | 5’-TTGCTAAAAATGATAGGGATGCT-3’ |
| PHL4-F | 5’-TCCAGCAACCAAGTATGCAATC-3’ |
| PHL4-R | 5’-GCGCAATTCCACACAAGAAGA-3’ |

**Supplementary Table 4.** Primers used for construction of the vectors used in LCI and BiFC assays

| **Primer name** | **Primer sequence** |
| --- | --- |
| PHL4-nluc-F-KpnI | 5’-GAGAACACGGGGGACGAGCTCGGTACC ATGATCCCGAATGATGATGATG-3’ |
| PHL4-nluc-R-SalI | 5’-GACGCGTACGAGATCTGGTCGAC  TTCTTCATTTCTCGGACGCTT-3’ |
| PHR1-cluc-F-KpnI | 5’-CTCGTACGCGTCCCGGGGCGGTACC  ATGGAGGCTCGTCCAGTTCAT-3’ |
| PHR1-cluc-R-SalI | 5’-CGAACGAAAGCTCTGCAGGTCGAC TCAATTATCGATTTTGGGACGC-3’ |
| PHL4-nYFP--F-KpnI | 5’-AGAACACGGGGGACGAGCTCGGTACC ATGATCCCGAATGATGATGATG-3’ |
| PHL4-nYFP--R-ApaI | 5’-GTCGAGTGAGGAGAAGAGCCGGGCCCC TTCTTCATTTCTCGGACGCTT-3’ |
| PHR1-cYFP--F-KpnI | 5’-TCTCGTACGCGTCCCGGGGCGGTACC ATGGAGGCTCGTCCAGTTCATA-3’ |
| PHR1-cYFP--R-SalI | 5’-CAACATATCCAGTCACTATGGTCGAC TCAATTATCGATTTTGGGACGC-3’ |

**Supplementary Table 5.** Primers used for construction of the vectors used in EMSA

| **Primer name** | **Primer sequence** |
| --- | --- |
| P1BS F | 5’-ATTAACGAATATTCCGTACA ATTAACGAATATTCCGTACA-3’ |
| P1BS R | 5’-TGTACGGAATATTCGTTAATTGTACGGAATATTCGTTAAT-3’ |
| P1BS F-biotin | 5’-ATTAACGAATATTCCGTACAATTAACGAATATTCCGTACA-3’ |
| P1BS R-biotin | 5’-TGTACGGAATATTCGTTAATTGTACGGAATATTCGTTAAT-3’ |
| pMAL-PHL4-NotI-F | 5’-CACATATGTCCATGGGCGGCCGC ATGATCCCGAATGATGATGATG-3’ |
| pMAL-PHL4-SalI-R | 5’-GCAGGGAATTCGGATCCGTCGAC-3’  TCAGTGGTGGTGGTGGTGGTG TTCTTCATTTCTCGGACGC-3’ |
