# Supplementary material for: Functional Characterization of Arabidopsis PHL4 in Plant Response to Phosphate Starvation
Source: Front Plant Sci. 2018 Oct 1;9:1432. doi: 10.3389/fpls.2018.01432 (PMC6174329; doi:10.3389/fpls.2018.01432)
Supplement: Supplementary file 2 [file Data_Sheet_1.PDF]

Supplementary figures

Functional Characterization of Arabidopsis PHL4 in Plant Response to Phosphate Starvation

Zhen Wang, Zai Zheng, Li Song, Dong Liu*

⋆ Correspondence:

Dong Liu, School of Life Sciences, Tsinghua University, Beijing 100084, China

Email: liu-d@tsinghua.edu.cn

Supplementary Figure 1

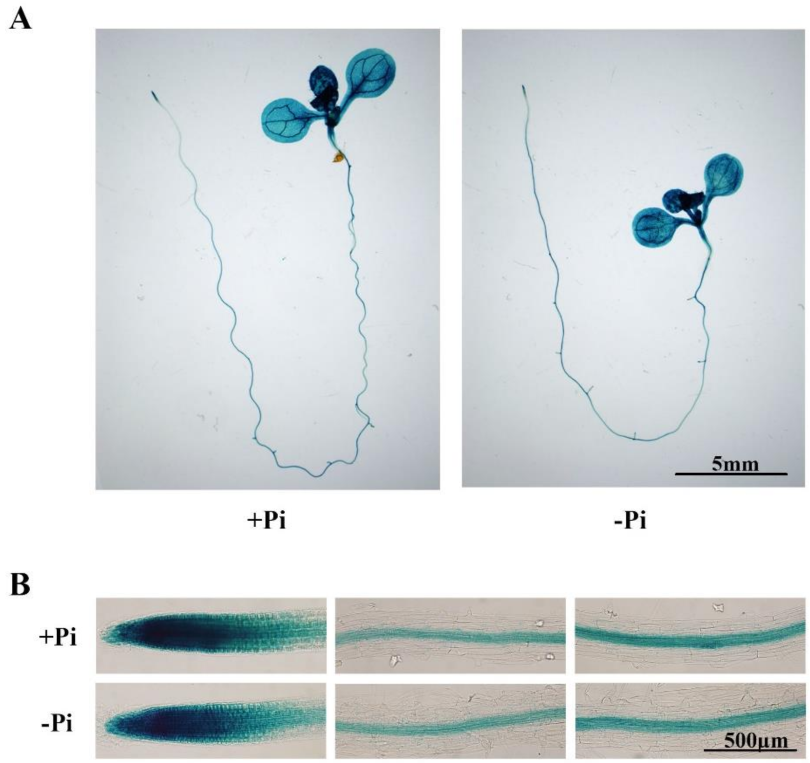

Supplementary Figure 1. Expression patterns of the *PHL4::GUS* on +Pi and –Pi media. **(A)** 8-day-old *PHL4::GUS* transgenic seedlings. **(B)** A close view of the root tips and maturation zones.

Supplementary Figure 2

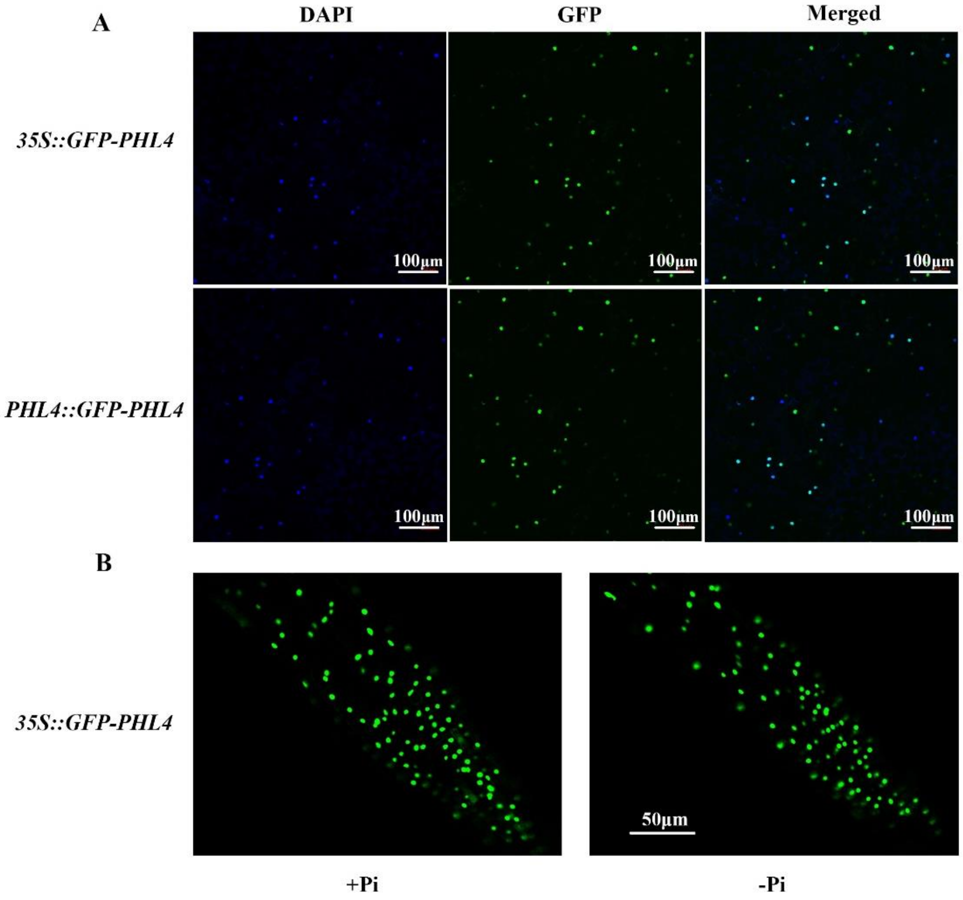

Supplementary Figure 2. Subcellular localization of GFP-PHL4 proteins. **(A)** Localization of GFP-PHL4 in the leaves of *N. benthamiana*. The expression of GFP-PHL4 was driven by *35S* promoter or the *PHL4* native promoter. The nuclei were stained with DAPI. GFP fluorescence was observed 2 days after infiltration. **(B)** Localization of GFP-PHL4 in the root tip of *35S::GFP-PHL4* Arabidopsis plants grown on +Pi and –Pi media.

Supplementary Figure 3

A

(a)

(b)

(c)

(d)

(e)

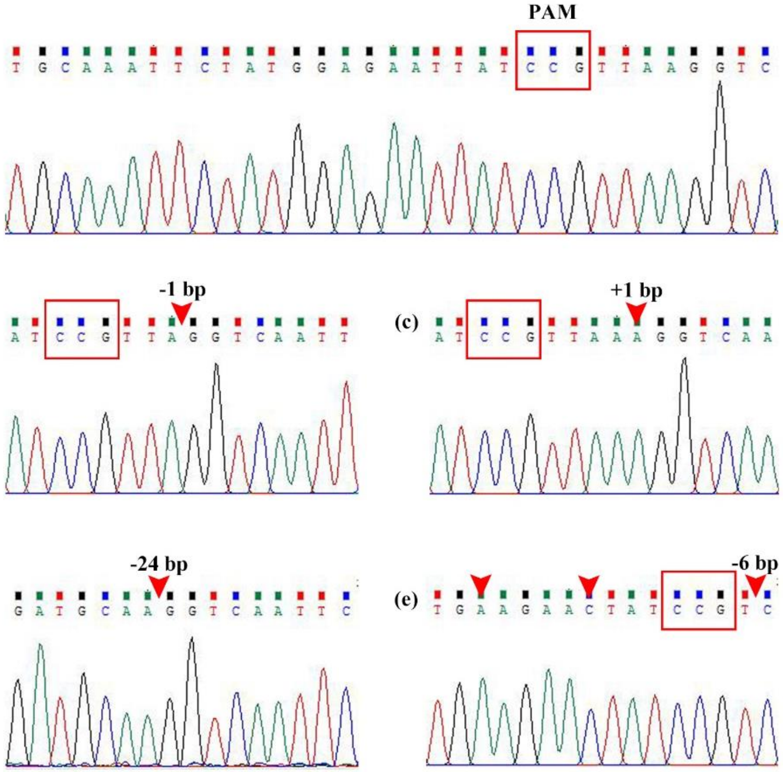

(f) 5'-GATGCAAATTCTATGGAGAATTATCCGTTAA--GGTCAATT-3' WT

5'-GATGCAAATTCTATGGAGAATTATCCGTTA---GGTCAATT-3' *phl4-1* (-1 bp)

5'-GATGCAAATTCTATGGAGAATTATCCGTTAAAGGTCAATT-3' *phl4-2* (+1 bp)

5'-GATGCAA ---GGTCAATT-3' *phl4-3* (-24 bp)

5'-GATGCAAATTCTATGAAGAACTATCCGT ---------------CAATT-3' *phl4-4* (-6 bp)

B

(a) MIPNDDDDANSMKNYPLNDDDANSMKNYPLNDDDANSMENYPLRSIPTELSHT
CSLIPPSLPNPSEAAADMSFNSELNQIMARPCDMLPANGGAVGHNPFLEPGFNC
PETTDWIPSPLPHIYFPSGSPNLIMEDGVIDEIHKQSDLPLWYDDLITTDEDPLM
SSILGDLLLDTNFNSASKVQQPSMQSQIQQPQAVLQQPSSCVELRPLDRTVSSNS
NNNSNSNNAAAAAKGRMRWTPELHEVFVDAVNQLGGSNEATPKGVLKHMKV
EGLTIFHVKSHLQKYRTAKYIPVPSEGSPEARLTPLEQITSDDTKRGIDITETLRI
QMEHQKKLHEQLESLRTMQLRIEEQGKALLMMIEKQNMGFGGPEQGEKTSA
KTPENGSEESESPRPKRPRNEE

(b) MIPNDDDDANSMKNYPLNDDDANSMKNYPLNDDDANSMENYPLRSIPTELSHT WT

MIPNDDDDANSMKNYPLNDDDANSMKNYPLNDDDANSMENYPLGQFRRSFHTLVH* *phl4-1*

MIPNDDDDANSMKNYPLNDDDANSMKNYPLNDDDANSMENYPLKVNSDGAFTHLFI
DTTFFTKPFRSSSRHVLQFRTQSNHGKAL* *phl4-2*

MIPNDDDDANSMKNYPLNDDDANSMKNYPLNDDDA ----------------RSIPTELSHT *phl4-3*

MIPNDDDDANSMKNYPLNDDDANSMKNYPLNDDDANSMKNYP----SIPTELSHT *phl4-4*

Supplementary Figure 3. Editing of the *PHL4* gene by CRISPR/Cas9 gene editing system. **(A)** DNA sequencing traces around the editing site of WT *PHL4* **(a)**, *phl4-1* **(b)**, *phl4-2* **(c)**, *phl4-3* **(d)**, and *phl4-4* **(e)**. The PAM sequence CCG is shown in red box. The positions where a deletion, insertion, or conversion of nucleotide(s) are indicated by red triangles. **(f)**: Alignment of the DNA sequences around the editing site among WT PHL4 and four *phl4* alleles. The PAM sequence is shown in pink. An inserted "A" in *phl4-1* is shown in red. The two substituted nucleotides in *phl4-4* are shown in blue. **(B)** The changes of the amino acid sequence in PHL4 in four *phl4* alleles resulted from the gene editing. **(a)** The amino acid sequence of WT PHL4. The MYB domain is shown in red and the coiled-coil domain is shown in blue. **(b)** Alignment of the amino acid sequences around the editing sites among the WT PHL4 and four mutated PHL4 proteins. The altered amino acid sequences in *phl4-1* and *phl4-2* caused by frameshift are shown in red. An asterisk indicates a stop codon. The deleted amino acid sequences in *phl4-3* and *phl4-4* are indicated by a dash line. A substituted amino acid in *phl4-4* is shown in blue.

Supplementary Figure 4

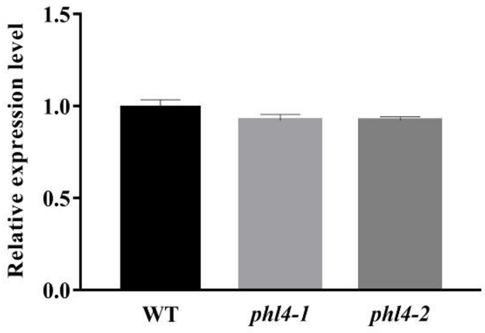

Supplementary Figure 4. The expression level of *PHL4* in the WT and two mutant alleles. 8-day-old seedlings grown on +Pi media were used for qPCR analysis. Values are the means ± SD of three biological replicates and represent fold changes normalized to transcript levels of *PHL4* in the WT grown on +Pi medium.

Supplementary Figure 5

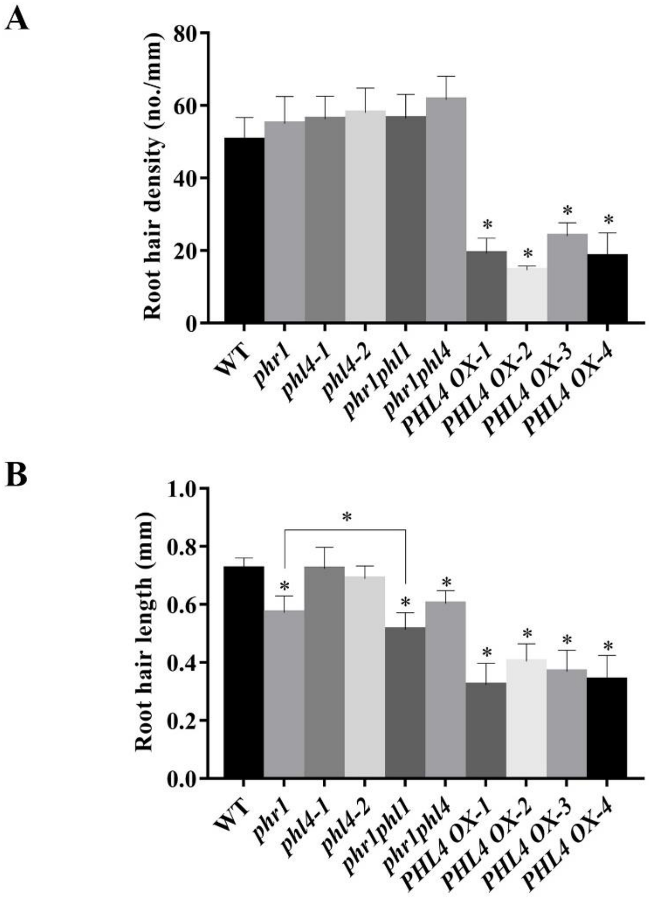

Supplementary Figure 5. Quantitative analyses of root hair density and root hair length of the WT, *phr1*, *phl4-1*, *phl4-2*, *phr1phl1*, *phr1phl4*, and four *PHL4 OX* lines for 6 days after germination. **(A)** Root hair density. Values represent means ± SD of 10 roots with 1 mm in length. **(B)** Root hair length. Values represent means ± SD of 10 roots (each root used 10 root hairs). In **(A)** and **(B)**, Asterisks indicate a significant difference from the WT ($P < 0.05$, *t*-test). Means with asterisks and a thin line are significantly different from each other ($P < 0.05$, *t*-test).

Supplementary Figure 6

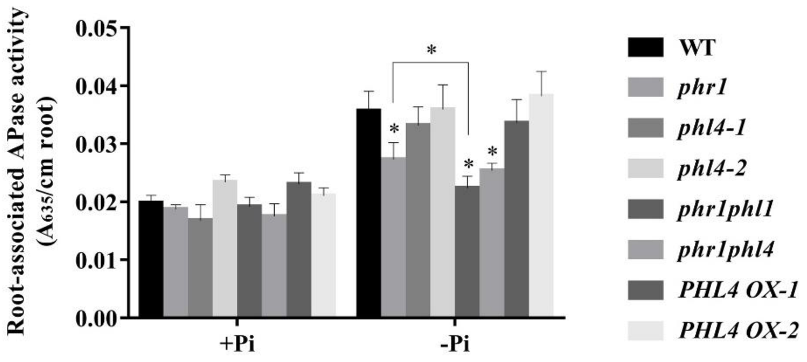

Supplementary Figure 6. Root-associated APase activity of WT, *phr1*, *phl4-1*, *phl4-2*, *phr1phl1*, *phr1phl4*, and four *PHL4 OX* lines for 6 days after germination. Values represent means ± SD of five replicates. Asterisks indicate a significant difference from the WT ($P < 0.05$, *t*-test). Means with asterisks and thin lines are significant different from each other ($P < 0.05$, *t*-test).
